# Response to three doses of the Pfizer/BioNTech BNT162b2 COVID-19 vaccine: a retrospective study of a cohort of haemodialysis patients in France

**DOI:** 10.1186/s12882-022-02751-5

**Published:** 2022-05-18

**Authors:** Jean-François Verdier, Sonia Boyer, Florence Chalmin, Ahmed Jeribi, Caroline Egasse, Marie France Maggi, Philippe Auvray, Tarik Yalaoui

**Affiliations:** 1 Centre de Néphrologie d’Antibes/Centre d’Hémodialyse de la Riviera, 103 ter avenue de Nice, F-06600 Antibes, France; 2 Laboratoire Bioesterel, F-06600 Antibes, France; 3 B.Braun Medical SAS, F-92210 Saint-Cloud, France

**Keywords:** SARS-CoV-2, COVID-19, Haemodialysis, Vaccine, Comirnaty

## Abstract

**Background:**

The mortality rate associated with coronavirus disease 2019 (COVID-19) is high among haemodialyzed patients. We sought to describe the serological status of haemodialysis patients having received up to three doses of BNT162b2 mRNA vaccine, and to identify factors associated with a poor humoral response.

**Methods:**

We performed a retrospective, observational study of patients attending a dialysis centre in Antibes, France. One or two of each patient’s monthly venous blood samples were assayed for anti–spike (S1) immunoglobulin G (IgG).

**Results:**

We included 142 patients, of whom 124 remained COVID-19-negative throughout the study. Among these COVID-19-negative patients, the humoral immune response rate (defined as an anti-S1 IgG titre ≥1.2 U/ml) was 82.9% after two injections and 95.8% after three injections, and the median [interquartile range] titre increased significantly from 7.09 [2.21; 19.94] U/ml with two injections to 93.26 [34.25; 176.06] U/ml with three. Among patients with two injections, the mean body mass index and serum albumin levels were significantly higher in responders than in non-responders (26.5 kg/m^2^ vs. 23.2 kg/m^2^, *p* = 0.0392; and 41.9 g/l vs. 39.0 g/l, *p* = 0.0042, respectively). For the study population as a whole at the end of the study, a history of COVID-19, at least two vaccine doses, and being on the French national waiting list for kidney transplantation were the only factors independently associated with the anti-S1 IgG titre.

**Conclusions:**

Dialysis patients vaccinated with two doses of BNT162b2 might not be sufficiently protected against SARS-CoV-2 and so should receive a third (booster) dose.

**Trial registration:**

The present retrospective study of clinical practice was not interventional and so was not registered.

## Background

The emergence of a novel form of infectious respiratory disease in late 2019 has since led to a global pandemic of coronavirus disease 2019 (COVID-19) and millions of death worldwide. In the absence of effective anti-infective medications and the relative inability of containment measures to stop the spread of the pathogenic severe acute respiratory syndrome coronavirus 2 (SARS-CoV-2) on the population level, public health strategies have relied on mass (voluntary) vaccination. Fortunately, the vaccines developed, tested clinically and approved in 2020 have given very encouraging results in terms of preventing clinical cases (and especially severe cases) of COVID-19 among vaccinated individuals [1].

Most countries have adopted a priority-based system to mass vaccination [2]. Given the high COVID-19 mortality rates observed in patients with chronic kidney disease (CKD) in general and those receiving renal replacement therapy in particular [3, 4], the nephrology community has emphasized the need to prioritize vaccination for patients [5, 6]. It must be borne in mind that the pivotal clinical trials required for marketing authorization of the forerunner vaccines did not include patients with physician-diagnosed CKD [7]. In France, patients on haemodialysis became eligible for vaccination with approved mRNA vaccines (BNT162b2 (Comirnaty) from Pfizer/BioNtech, or mRNA-1273 from Moderna) on January 18th, 2021 (https://solidarites-sante.gouv.fr/IMG/pdf/dgs_urgent_04_vaccination_patients_a_risque.pdf). However, the initially published results on the immune response to COVID-19 vaccination in haemodialysis patients were inconsistent: some studies evidenced a weak (subnormal) response, whereas others evidenced an essentially normal response, with high titres of IgG against SARS-CoV-2’s spike (S1) protein [8–14]. These disparate findings (for reviews, see [15, 16]) prompted us to investigate the vaccination rate and the vaccine response in a cohort of haemodialysis patients attending our dialysis centre in Antibes, France.

The primary objective of the present retrospective, real-life study was to describe the serological status of haemodialysis patients after vaccination with BNT162b2, with a focus on three doses vs. two doses. The secondary objective was to identify factors associated with a poor response to COVID-19 vaccination, in order to refine the advice given to our patients.

## Methods

### Study design

**Fig. 1:**
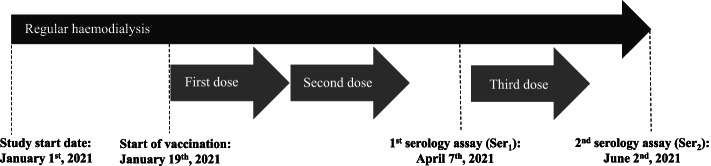
Study timeline. It should be noted that not all participants received three doses of vaccine, and that the vaccination dates varied from one participant to another. Hence, the figure shows the sequence of events for a typical participant with three doses and two serology assays

We performed a retrospective analysis of routine medical data from haemodialysis patients attending a single dialysis centre in Antibes (France) owned by B. Braun Avitum GIE (Saint-Cloud, France). In line with the French guidelines, we encouraged patients attending the centre to undergo vaccination with two doses of BNT162b2 administered at least 3 weeks apart (Fig. 1). In view of the response to two doses (see below), we then recommended a third (booster) dose of vaccine.

All haemodialysis patients attending our centre undergo a standard venous blood screen (sample volume: 3.5 ml) on a monthly basis. Serology assays (see below) were performed on the blood samples collected on April 7th, 2021 (after 105 out of 126 (83.3%) documented COVID-19-negative patients had received two doses of vaccine), and June 2nd, 2021 (after 96 out of 120 (80.0%) documented COVID-19-negative patients had received three doses of vaccine). The study inclusion period ran from January 19th, 2021 (the date of the first vaccination of an included patient) and June 2nd, 2021 (the date of the last serology assay for an included patient).

### Patient selection

The main inclusion criteria were (i) attendance at the dialysis centre between January 1st and June 2nd, 2021, (ii) the availability of standard laboratory data on monthly blood samples (starting no later than April, 2020), (iii) a time interval of at least 21 days between consecutive doses of BNT162b2, and (iv) the availability of data on SARS-CoV-2 serology at least 21 days after the last dose of BNT162b2.

Personal medical data (including previous and ongoing medical conditions and medications) were extracted from the patients’ medical records. The effectiveness of dialysis (removal of urea in the dialysate) was defined as the Kt/V, as measured with the Adimea module on BBraun Dailog plus or BBraun IQ systems (B Braun Melsungen AG, Melsungen, Germany). All the study participants were vaccinated by the centre’s nurses. The presence of a supervising physician in the dialysis centre was compulsory.

### Immunoglobulin assays

The patients’ plasma anti-S1 IgG titre and thus serological status was determined using a chemiluminescence-based SARS-CoV-2 IgG Assay running on a Atellica® IM system (both from Siemens Healthcare GmbH, Erlangen, Germany), according to the manufacturer’s instructions. The assay results are quoted in U, which (according to Siemens) corresponds to 21.8 binding antibody units (BAU) [17]. The assay has been validated, and the anti-S1 titre shows a good correlation with virus neutralization titres [18, 19]. According to a report by Pflüger et al., the Siemens SARS-CoV-2 IgG assay has a specificity [95% confidence interval] of 100% [98.8–100], a positive predictive value of 100% (calculated 10 days after a positive polymerase chain reaction (PCR) test, for a seroprevalence of 0.8%) and a negative predictive value of 99.8% [20]. According to the manufacturer, the threshold for a positive anti-S1 IgG titre in the Siemens SARS-CoV-2 IgG Assay is 1.2 U/ml [21]. Individuals with a titre between 0.8 and 1.2 U/ml are classified as “borderline”, and a titre below 0.8 U/ml indicates the absence of a response [21]. Hence, we defined vaccine non-responders as patients with an anti-S1 IgG titre below 0.8 U/ml after having received two or more doses of BNT162b2. Vaccine responders were defined as patients with an anti-S1 IgG titre above 1.2 U/ml after having received two or more doses of BNT162b2.

### Ethics

In line with the French legislation on re-analyses of routinely collected medical data, approval by an independent ethics committee was neither required nor sought. In our centre, regular serological and/or PCR testing of dialyzed patients for infectious (viral) diseases is standard practice. For example, our patients are tested for HIV twice a year. The participants were given information on the study’s procedures and objectives, and were provided with their SARS-CoV-2 PCR and anti–S1 antibody results. All the participants confirmed that they did not object to the processing of their personal medical data for the purposes of the present study. The study was performed in compliance with the MR-004 benchmark methodology (https://www.legifrance.gouv.fr/jorf/id/JORFTEXT000037187443) specified by the French National Data Protection Commission (*Commission nationale de l’informatique et des libertés*, Paris, France).

### Statistical analysis

Continuous variables were described as the mean (standard deviation (SD)), median [interquartile range (IQR)], and range. Categorical variables were described as the frequency (percentage). The statistical significance of intergroup differences was assessed with Fisher’s exact test (for categorical variables) or the Kruskal-Wallis test (for continuous variables). The threshold for statistical significance was set to *p* < 0.05.

The relationship between the anti-S1 IgG titre and patient characteristics was tested in a univariate linear regression analysis with the following factors: age, sex, body mass index (BMI: weight (kg)/height (m)^2^), diabetes, immunosuppressive treatment, being on the French national waiting list for kidney transplantation, time on dialysis, the blood lymphocyte count, the blood leukocyte count, Kt/V, and the serum albumin level. A multivariate model was then used to identify factors independent associated with the anti-S1 IgG titre. The “COVID-19 vaccination” variable was forced into the model. Next, variables with *p* < 20% in the univariate analysis were included in the model one by one, starting with the most significant. The multivariate model was finalized when the last parameter included was not significant on the *p* < 0.05 level. The anti-S1 IgG titres were square-root-transformed, in order to approximate a normal data distribution and to facilitate graphic comparisons.

The analysis was repeated for each of the two serology assay time points. For the analysis of the square root of the anti-S1 IgG titre vs. the number of doses of vaccine received (in a linear mixed model), the two serology assay time points were pooled. Hence, two values were available for most patients. To take account of the clustering of the serology results for each patient, a random effect was included in the model for the “patient” variable.

The correlation between the square root of the anti-S1 IgG titre on one hand and age and the square root of the anti-hepatitis B titre was assessed by calculating Spearman’s coefficient. All statistical analyses were performed using SAS software (version 9.4, SAS Institute, Inc., Cary, NC).

## Results

### Characteristics of the study population

**Fig. 2:**
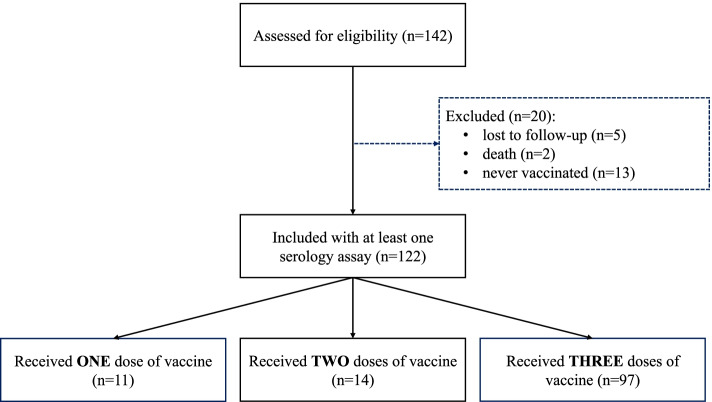
Study flow chart

A total of 142 patients had at least one anti-S1 IgG serology value in April or June 2021 and were included in the study (Figs. 1 and 2, and Table 1). All but one of the patients were seronegative for anti-S1 IgG in an assay performed 7 months before the start of the study (in May 2020). However, the one seropositive patient was asymptomatic. As expected for a population of patients on dialysis, most of the participants were older adults (mean age: 71.1), with a majority of men (*n* = 103; 72.5%).

**Table 1:** Demographic and clinical characteristics of the study population. All data are reported for *n* = 142 participants at the time of the first serology assay, unless otherwise stated

Variable	Value
Age (years)	
Mean (SD)	71.1 (13.1)
Median [IQR]	74.0 (64.0; 81.0)
Range	(32.0; 93.0)
Sex, n (%)	
Females	39 (27.5%)
Males	103 (72.5%)
BMI (kg/m^2^)	
Mean (SD)	25.3 (5.5)
Median [IQR]	24.2 (21.7; 27.4)
Range	(14.4; 54.2)
Diabetes, n (%)	61 (43.0)
Immunosuppressant treatment, n (%)	21 (14.8)
On the French national waiting list for kidney transplantation, n (%)	48 (33.8)
Time on dialysis (months)	
Mean (SD)	69.0 (93.4)
Median [IQR]	34.6 (11.1; 85.2)
Range	(0.2; 439.2)
Kt/V	
Mean (SD)	1.5 (0.3)
Median [IQR]	1.5 (1.3; 1.7)
Range	(0.6; 2.2)
Time interval between the first vaccine dose and Ser_1_ (*n* = 126)	
Mean (SD)	62.2 (28.9)
Median [IQR]	75.0 (71.0; 76.0)
Range	(−4.0; 78.0)
Time interval between the second vaccine dose and Ser_1_ (*n* = 115)	
Mean (SD)	45.7 (18.1)
Median [IQR]	50.0 (50.0; 54.0)
Range	(− 55.0; 57.0)
Time interval between the third vaccine and Ser_2_ (*n* = 100)	
Mean (SD)	35.0 (0.1)
Median [IQR]	35.0 (35.0; 35.0)
Range	(35.0; 36.0)
Leukocyte count (× 10^9^/L)	
Mean (SD)	7.4 (2.7)
Median [IQR]	7.0 (5.5; 8.7)
Range	(2.5; 20.7)
Lymphocyte count (× 10^6^/L)	
Mean (SD)	1573.7 (1149.2)
Median [IQR]	1410.0 (1049.0; 1821.0)
Range	(242.0; 12,479.0)
Serum albumin (g/l)	
Mean (SD)	41.3 (3.3)
Median [IQR]	41.0 (39.0; 44.0)
Range	(32.0; 48.0)

*SD* Standard deviation, *IQR* Interquartile range, *BMI* Body mass index, *Kt/V* Dialysis rate, *Ser*_*1*_ First serology assay, *Ser*_*2*_ Second serology assay

Eleven patients had a positive PCR test for SARS-CoV-2 RNA at the start of the retrospective study period (January 19th, 2021) and so were not vaccinated. Between the start of the study and the first serology assay (henceforth referred to as Ser_1_) on April 7th, 2021, a further 5 patients became COVID-19-positive (4 doubly vaccinated patients and 1 non-vaccinated patient). Lastly, 2 patients became COVID-19-positive (one doubly vaccinated patient and one non-vaccinated patient) between the first serology assay and the second serology assay. Overall, 13 patients were never vaccinated (due variously to COVID-19 infection, refusal, or allergy to a vaccine component) and were excluded from the analysis.

The data for Ser_1_ concerned patients having received one dose of vaccine (*n* = 3) or two doses (*n* = 111) at that point in time. The data from the second serology assay (on June 2nd, 2021, henceforth referred to as Ser_2_) concerned patients having received one dose of vaccine (*n* = 11), two doses (*n* = 14) or three doses (*n* = 97). On average, the first and second doses of vaccine were given respectively 62 and 46 days before Ser_1_. The third dose was given 35 or 36 days before Ser_2_.. No unexpected adverse events associated with vaccination were reported by the vaccinated patients or the centre’s medical staff.

### The anti-S1 IgG titres for one and two doses of vaccine

At Ser_1_, 105 out of 126 (83.3%) documented COVID-19-negative patients had received two doses of vaccine. At Ser_1_, a response to vaccine (i.e. an anti-S1 IgG titre ≥1.2 U/ml) was observed in 87 (82.9%) of the 105 COVID-19-negative patients with two injections. In a comparison of non-responders and responders, we found that the mean BMI and the mean serum albumin levels were slightly but significantly higher in responders than in non-responders (26.5 kg/m^2^ vs. 23.2 kg/m^2^, *p* = 0.0392; and 41.9 g/l vs. 39.0 g/l, *p* = 0.0042, respectively). These were the only significant differences in anthropomorphic, laboratory and clinical variables between the responders and non-responders.

It is noteworthy that at Ser_1_, the 10 COVID-19-positive, non-vaccinated patients had higher mean and median titres (22.0 and 15.5 U/ml, respectively) than the 108 patients having received one or two doses (18.5 and 6.8 U/ml, respectively); the difference was not significant (*p* = 0.0587) but this may have been due to the large difference between the two sample sizes (10 and 108).

### The anti-S1 IgG titres for two vs. three doses of vaccine

**Fig. 3:**
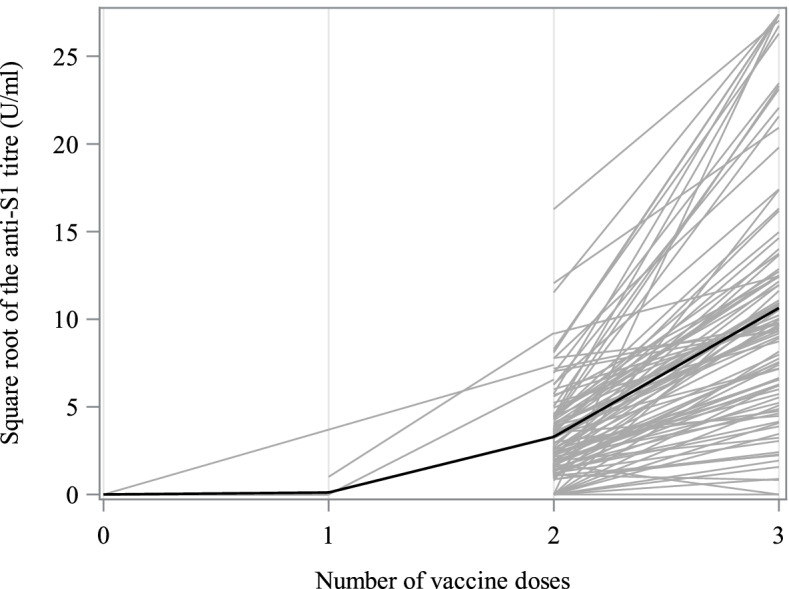
Individual anti-S1 Ig titres for COVID-19-negative patients. Data are shown as a function of the number of doses of vaccine received. Each grey line represents the change in an individual patient’s titre between the first and second serology assays (Ser_1_ and Ser_2_). The thick, black line was derived by non-parametric, locally estimated smoothing; it illustrates the trend for the group

The large, dose-dependent increases in the individual anti-S1 Ig titre and the non-optimal response rate prompted us to recommend a third dose to our patients (Tables 2 and 3, Fig. 3). At Ser_2_, 96 of the 120 (80%) documented COVID-19-negative patients had received three doses of vaccine; a response to vaccine was observed in 92 of the 96 (95.8%).

**Table 2:** Anti-S1 IgG titres and response class at the end of the study

Variable	Value
Anti-S1 IgG (participants with three doses of vaccine), U/ml (*n* = 97; all but one were COVID-19-negative in a PCR test)	
mean (SD)	170.06 (212.12)
median [IQR]	93.26 [34.25; 176.06]
range	(0.0; 750.0)
Anti-S1 IgG (COVID-19-positive participants), U/ml (*n* = 15)	
mean (SD)	351.4 (352.0)
median [IQR]	294.9 (4.2; 750.0)
range	2.5; 750.0
Response class in COVID-19-negative patients with two doses of vaccine, n (%) (*n* = 7)	
Non-responders (< 0.8 U/ml)	0
Borderline (0.8 to 1.2 U/ml)	2 (28.6%)
Responders (≥1.2 U/ml)	5 (71.4%)
Response class in COVID-19-negative patients with three doses of vaccine, n (%) (*n* = 96)	
Non-responders (< 0.8 U/ml)	0
Borderline (0.8 to 1.2 U/ml)	4 (4.2%)
Responders (≥1.2 U/ml)	92 (95.8%)
Response class in patients with three doses (whatever the COVID-19 status), n (%) (n = 97)	
Non-responders (< 0.8 U/ml)	0
Borderline (0.8 to 1.2 U/ml)	4 (4.1%)
Responders (≥1.2 U/ml)	93 (95.9%)

*S1* Spike, *IgG* Immunoglobulin G, *COVID-19* Coronavirus disease 2019, *PCR* Polymerase chain reaction, *SD* Standard deviation, *IQR* Interquartile range

**Table 3:** Anti–spike 1 immunoglobulin G titres in COVID-19-negative patients, by the number of vaccine injections

Number of patients	0 injection	1 injection	2 injections	3 injections
Ser_1_	18	3	105	0
Ser_2_	11	6	7	96
Total	29	9	112	96
Mean (SD) anti-S1 Ig titre (U/ml)	0 (0)	0.11 (0.34)	19.5 (34.4)	170.06 (212.12)
Median [IQR] anti-S1 Ig titre (U/ml)	0 [0; 0]	0 [0; 0]	7.09 [2.21; 19.94]	93.26 [34.25; 176.06]

*Ser*_*1*_ First serology assay, *Ser*_*2*_ Second serology assay, *SD* Standard deviation, *IQR* Interquartile range, *anti-S1 Ig* Anti–spike 1 immunoglobulin G

We next analyzed the data as a function of the number of doses of vaccine received by COVID-19-negative patients (Table 3). The mean (SD) titre rose from 19.5 (34.4) U/ml after two doses of vaccine to 170.06 (212.12) U/ml after three doses (425 and 3707 BAU/ml, respectively), and the median [IQR] titre rose from 7.09 [2.21; 19.94] U/ml to 93.26 [34.25; 176.06] U/ml (154 and 2033 BAU/ml, respectively).

### Factors associated with the anti-S1 IgG titres

**Fig. 4:**
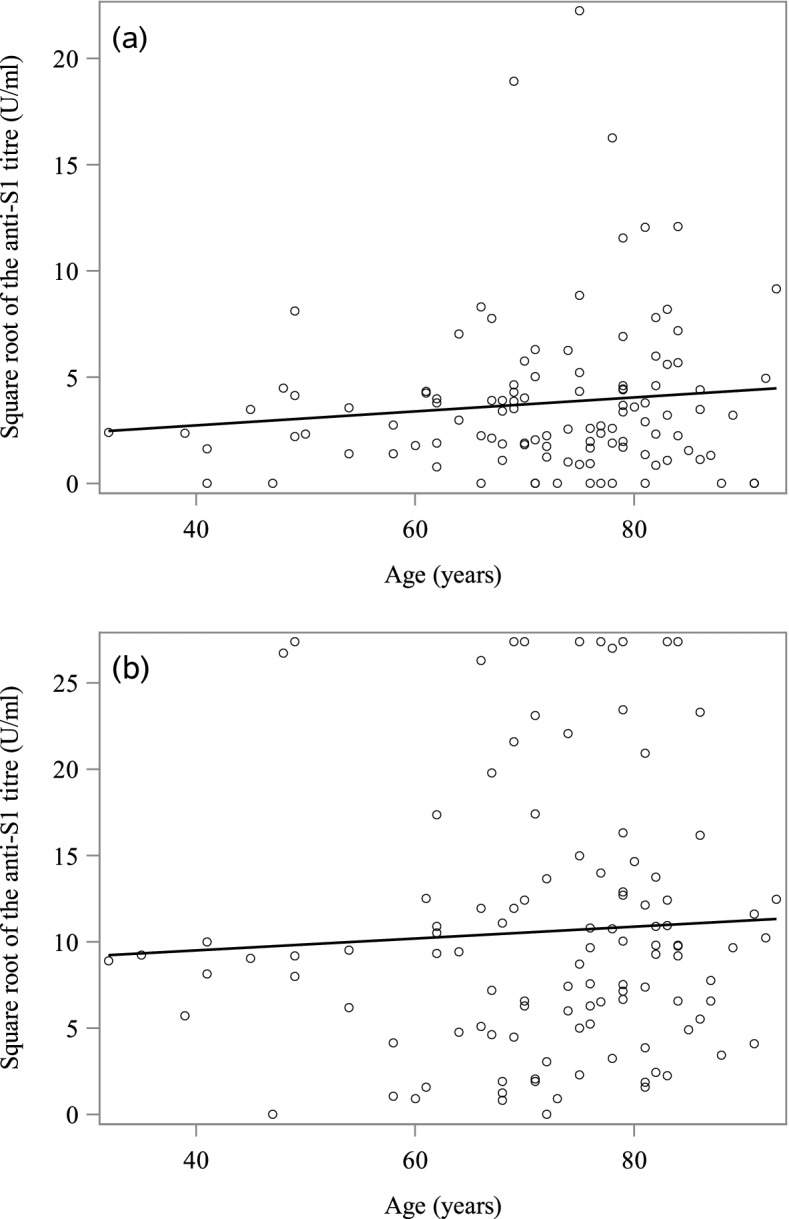
Correlation between the anti-S1 Ig titre and age. Data are shown for patients with two or three doses of vaccine (*n* = 111) for the first (**a**) and second (**b**) serology assays

In a multivariate analysis at Ser_1_, five factors were found to be independently and significantly associated with the anti-S1 IgG titre: a history of COVID-19, the receipt of at least two doses of vaccine, being on the French national waiting list for kidney transplantation, and female sex were associated with a higher anti-S1 IgG titre, whereas immunosuppressive treatment was associated with a lower anti-S1 IgG titre. In our patients, the indications for treatment with immunosuppressive factors were transplantation (cyclosporine, sirolimus, tacrolimus, etc.) and chronic inflammatory diseases (immunotherapies, corticoids, etc.). In a multivariate analysis at Ser_2_, only three factors were found to be independently and significantly associated with the anti-S1 IgG titre (Table 4): a history of COVID-19, the receipt of at least two doses of vaccine, and being on the French national waiting list for kidney transplantation. Hence, neither age nor immunosuppressive treatment was significantly associated with the anti-S1 IgG titre at Ser_2_. In patients with at least two doses of vaccine, Spearman’s correlation coefficient r was 0.07 (*p* = 0.4970) at Ser_1_ and 0.11 (*p* = 0.2618) at Ser_2_ (Fig. 4).

**Table 4:** Factors associated with the anti-S1 IgG titre at the end of the study

Variable	DF	Parameter estimate	Standard error	t Value	Pr > |t|
Intercept	1	1.41916	1.71706	0.83	0.41
A history of COVID-19	1	7.84042	2.14416	3.66	0.0004
At least two doses of vaccine	1	7.42861	1.75461	4.23	<.0001
Being on the French national waiting list for kidney transplantation	1	3.77334	1.38702	2.72	0.0074

The multivariate analysis covered all patients, regardless of their COVID-19 status. *N* = 135 patients, *p* < 0.0001 in a linear mixed model; R^2^ adjusted = 0.19. DF: degrees of freedom. Antibody titres were square-root-transformed

**Fig. 5:**
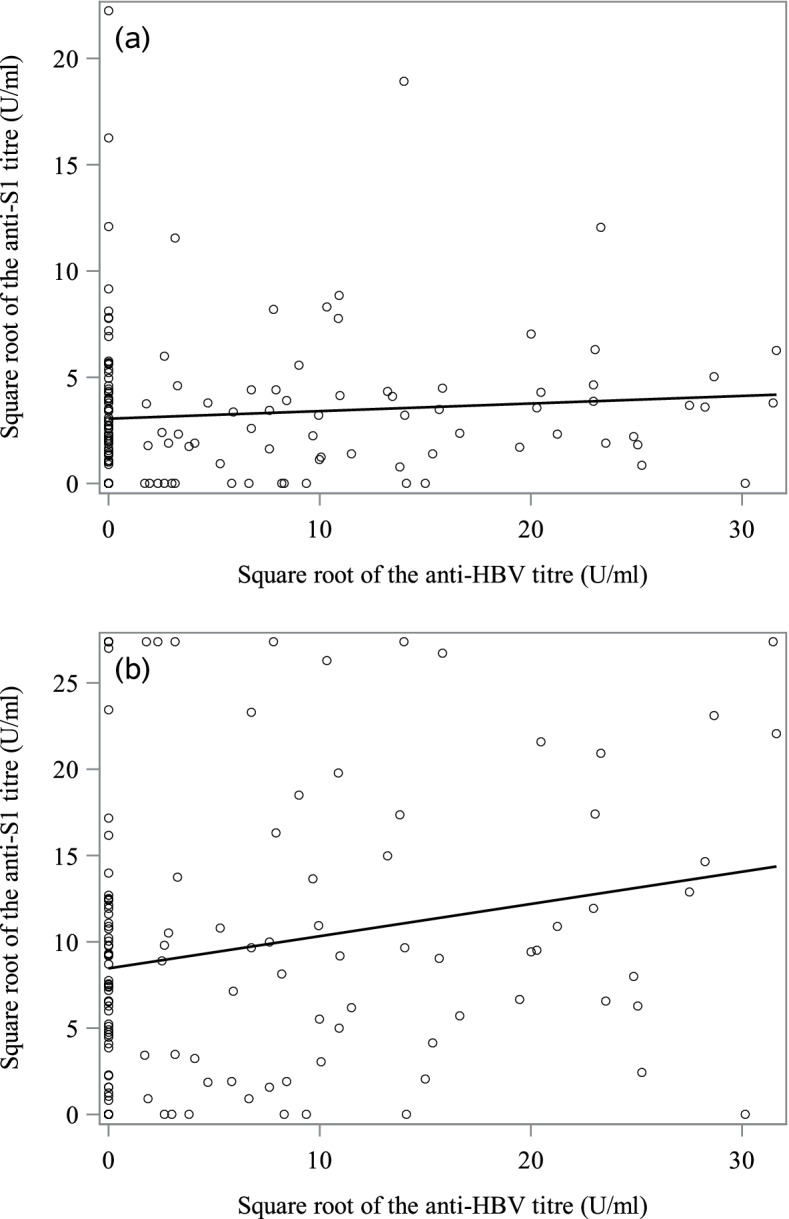
Correlation between the anti-S1 Ig titre and the anti-HBV titre. Data are shown for the first (**a**) and second (**b**) serology assays

Lastly, the correlation between the anti-HBV titre (measured on March 3rd, 2021) and the anti-S1 IgG titre was not statistically significant at Ser_1_ (when 83.3% of the COVID-19-negative patients had received two doses; Spearman’s correlation coefficient *r* = 0.12, *p* = 0.1438) but was significant at Ser_2_ (when 80.0% of the COVID-19-negative patients had received three doses; Spearman’s correlation coefficient *r* = 0.17, *p* = 0.0452) (Fig. 5).

## Discussion

The main findings of the present retrospective study of haemodialysis patients attending a single dialysis centre in France were as follows: (i) two doses of BNT162b2 were not enough for a robust humoral immune response in all our patients on dialysis, (ii) the anti-S1 IgG titres increased significantly after a third dose of vaccine (as would be expected for a “booster” injection), (iii) the mean BMI and the mean serum albumin levels were slightly but significantly higher in responders than in non-responders with two doses, (iv) three factors (a history of COVID-19, the receipt of at least two doses of vaccine, and being on the French national waiting list for kidney transplantation) were found to be independently and significantly associated with the anti-S1 IgG titre at the end of the study, (v) the anti-S1 IgG titre at the end of the study was not correlated with age or immunosuppressant treatment, and (vi) the anti-S1 IgG titre was correlated with the anti-HBV titre after three doses of vaccine but not after two doses.

Our results for a group of 100 dialysis patients having received one, two or three doses of BNT162b2 are in line with a number of literature reports – some of which appeared after our analysis had been performed. Firstly, with regard to the difference between one dose and two doses, Attias et al. found that the seropositivity rate was 18% before the second injection and 82% afterwards [8]. Grupper et al. reported that patients (*n* = 56) on maintenance haemodialysis developed a substantial humoral response following vaccination with BNT162b2 but that the anti-S1 IgG titres of against SARS-CoV-2’s spike (S1) protein (measured a median of 30 days after the second dose) were significantly lower than in a group of (younger) healthy controls [11]. However, a report by Torregiani et al. suggested that only about one-third of patients on haemodialysis developed neutralizing antibodies after the first dose of BNT162b2 [13]. Simon et al. observed very low median anti-S1 titres in dialysis patients (*n* = 81) after two doses of BNT162b2 (171 U/ml, versus 2500 U/ml in healthy controls) [12]. Billany et al. reported that SARS-CoV-2 neutralizing antibodies against the receptor binding domain of the spike protein were not detectable in 19 out of 94 patients receiving maintenance haemodialysis and who had received the BNT162b2 or the AZD1222 (Oxford-AstraZeneca) vaccine (20.2%) [9]. In Israel, Yanay et al. reported a lower response rate to the vaccine, a lower anti–S1 antibody titre, and a higher rate of COVID-19 infection after vaccination in a group of dialysis patients, relative to controls [14]. In a study of 90 vaccinated HD patients (mean age: 69) of whom 19 (21%) had a history of SARS-CoV-2 infection, Giot et al. documented anti-S IgG seroconversion in 20% of patients after the first dose and in 77% after the second dose [22]. In Nacasch et al.’s study, 19% of double-vaccinated haemodialysis had low or undetectable antibody levels [23].

With regard to the difference between two doses and three doses, Ducloux et al.’s study of a group of 50 dialysis patients observed a vaccine response rate of 90% among those having received two doses of BNT162b2 [10]. The administration of a third dose enhanced the antibody titre markedly in almost all patients and especially in those with low titres after two doses. Marked increases in the anti-S1 antibody titre were also reported by Francken et al. after the administration of a third dose of BNT162b2 to a subset of patients with a value below 250 U/ml (according to the Roche Diagnostic Elecsys® enzyme immunoassay) after two doses [24]. In a follow-up report on the patients studied by Giot et al., Robert et al. notably described the response to a third dose in 10 “partial responders” (defined as positive for circulating anti-S1 IgGs but negative for neutralizing antibodies after two doses) [25]. After a third dose, 7 of the 10 partial responders still had anti-S1 IgGs and four had developed neutralizing antibodies. The median [IQR] anti-S1 IgG titre was 31.5 [17.8–41.8] BAU/mL after two doses and 776.7 [138.3–3038] after three. Like Robert et al., Frantzen et al. chose to administer a third dose of BNT162b2 only when the individual titre was below a particular threshold [24]. Bensouna et al. found that a third dose of BNT162b2 increased antibody levels substantially in patients on maintenance dialysis and appeared to be as well tolerated as the second dose [26]. Lastly, Stumpf et al. reported on the humoral and cellular immune responses to boost vaccination with mRNA vaccines in a cohort of dialysis patients, kidney transplant patients and medical personnel; after the boost, the seroconversion efficacy among dialysis patients (> 95%) was similar to that seen among medical personnel [27].

In contrast to studies in which only non-responders or partial responders were given a third dose, we recommended a third dose to all our patients in the spring of 2021. This was not long after the start of the vaccination campaign in France, at a time when a third dose was not being considered for general population. At the time of writing, a three-dose COVID-19 vaccination regimen is recommended by the French health authorities for all individuals over the age of 12, regardless of whether or not risk factors are present. Hence, our medical decision became a legal requirement; in France, members of the general population aged over 12 must receive a third dose if they want a valid “vaccine passport”.

The design of the present retrospective, observational study prevented us from determining the causal nature of relationships between the anti-S1 IgG titre on one hand and demographic, clinical and laboratory factors on the other. The only small (but statistically significant) differences between non-responders and responders after two doses of vaccine concerned the BMI and serum albumin level. However, the BMI and serum albumin values observed here were not unusual for a haemodialyzed population. Furthermore, two of the factors associated with anti-S1 IgG titre (a history of COVID-19 and being on the French national waiting list for kidney transplantation) are of little practical value with a view to increasing the response rate among vaccinated patients on haemodialysis. Although French and European guidelines are available, placement on the French national waiting list depends on each clinical team’s practices; these differ from one region of France to another and even from one centre to another in the same region [28].

In our patients with at least two doses of vaccine, age was not significantly associated with the anti-S1 IgG titre (as also observed by Irsara et al. [19]). In most literature reports, however, age is a major confounding factor in the reports on the response to COVID-19 vaccines. In the reports by Nacasch et al., Simon et al. and Grupper et al., greater age was associated with a lower anti-S1 IgG titre [11, 12, 23]. Jahn et al. found that almost all dialysis patients under the age of 60 showed an essentially normal response (compared with controls) to a second (but not first) dose of BNT162b2 (median [IQR] titre: 597.0 arbitrary units (AU)/mL [410.5; 800.0]; in contrast, dialysis patients over the age of 60 had significantly lower antibody titres (median [IQR] titre: 280.0 AU/mL (45.7; 477.0); *p* < 0.0001) [29]. However, the fact that our study population was quite elderly (median [interquartile range] age: 74 [64–81]) might explain the lack of a significant correlation. The moderate proportion of (often young) patients on the transplant list (33.8%) and/or the presence of contraindications to transplantation (e.g. immunosuppression and cancer) might also contribute individually or collectively to the lack of a significant association.

It is noteworthy that in our multivariate analysis, treatment with immunosuppressants was associated with lower anti-S1 IgG titre at Ser_1_ but not at Ser_2_. We note that according to Benotmane et al. 2021, kidney transplant recipients (rather than patients on dialysis, as in our study) treated with calcineurin inhibitors, mycophenolate mofetil, or steroids showed significantly lower anti-SARS-CoV-2 antibody titres after one and two doses of the Moderna mRNA-1273 vaccine [30]. In a study of dialysis patients and kidney transplant recipients, Stumpf et al. reported that the number of immunosuppressive drugs and the type (belatacept, mycophenolate mofetil-mycophenolic acid, and calcineurin inhibitors) were risk factors for seroconversion failure [27]. In a study of haemodialysis patients by Nacasch et al., long-term immunosuppressive therapy was the primary predictor of low antibody titres in double-vaccinated individuals (odds ratio: 30.4; *p* < 0.001) [23]. More generally, it is noteworthy that COVID-19 vaccination is not contraindicated in France for the major immunosuppressants (corticosteroids, methotrexate, azathioprine, mycophenolate mofetil, hydroxychloroquine, leflunomide, sulfasalazine, anti-tumour necrosis factor agents, anti-CD20 antibodies, and Janus kinase inhibitors); the benefits of vaccination – even when dampened by immunosuppressants – far outweigh the risks of non-vaccination.

According to the manufacturer of the in vitro assay used in the present study, an anti-S1 antibody titre of 7 U/ml is “neutralizing” for SARS-CoV-2 [17]. Anand et al. suggested a slightly higher value of 10 U/ml [31]. Among the 112 COVID-19-negative patients having received two doses of the BNT162b2 vaccine, the median anti-S1 antibody titre was 7.09 U/ml; this means that at least half of these patients had a titre below or barely above the supposedly neutralizing value and so were probably not sufficiently protected. In contrast, the median titre rose markedly after the third dose (to 93.26 U/ml), which probably corresponds to a good level of protection. More broadly, our results highlight the urgent need for reliable correlates of protection (CoP) in patient groups that have not been well represented in clinical trials of COVID-19 vaccines. A CoP is a measure of the immune response that is significantly correlated with protection against infection, disease and/or transmission in vaccinated individuals. The use of CoP may allow the prediction of clinical outcomes more rapidly than in clinical trials – especially for rapidly emerging new variants of SARS-CoV-2. However, CoP are difficult to define and may depend on the characteristics of each type of vaccine, including the antigen, the vector, the antigen presentation method, the presence or absence of adjuvants, and the vaccination regimen (number of doses) – all of which might affect the humoral response, the cellular response, or both. CoPs have not been clearly defined for SARS-CoV-2 vaccines in general, let alone in groups of vulnerable individuals like dialysis patients.

It will be interesting to (i) follow up changes over time in our patients’ IgG titres in particular and other markers of the humoral response in general, and (ii) compare these data with the literature data on the general population. Interestingly, there is evidence to suggest that dialysis patients mount a cellular immune response (albeit imperfectly) to SARS-CoV-2 [27, 32], which might help to compensate for a weaker humoral response. Lastly, novel variants of SARS-CoV-2 emerge frequently, and it will be essential to determine whether certain variants represent a particular risk for dialysis patients.

Given that we only administered the Pfizer/BioNTech BNT162b2 vaccine, we can only speculate about other vaccines on the basis of the literature data. At the time of our initial submission, there were few literature data on vaccines other than Pfizer/BioNTech, and most of the pivotal vaccine trials exclude participants on dialysis. Since then, a few studies have assessed the Moderna mRNA vaccine (mRNA-1273) in patients on dialysis. In a study of peritoneal dialysis patients having received the mRNA-1273 vaccine, Rodriguez-Espinosa et al. found that the mean ± SD anti-S1 IgG titre rose from 28.09 ± 52.2 after the first dose to 113.7 ± 56.9 after the second dose (a third dose was not described) [33]. Interestingly, Kaiser et al. performed a comparative study in haemodialysis patients: those vaccinated with mRNA-1273 showed higher anti-S titres than those vaccinated with BNT162b2 [34]. In their prospective, multicentre observational study of people vaccinated with mRNA-1273 or BNT162b2, Stumpf et al. found that the seroconversion rate for the anti-S titre was significantly higher in dialysis patients vaccinated with mRNA-1273 than (95%) than in those vaccinated with BNT162b2 (85%, *p* < 0.001) [27]. However, Hasmann et al. pointed out that according to Stumpf et al.’s data, the response rates were still significantly lower in dialysis patients than in controls [35].

The present study had several strengths. Firstly, it described the response to three doses of BNT162b2 in a well-documented, relatively large (*n* = 100) group of patients on dialysis. Secondly, it provided comparative data on patients having received one, two and three doses of the vaccine. Thirdly, we identified factors associated with a lack of response to one and two doses of BNT162b2. The study also had a number of limitations, most of which were inherently associated with its retrospective design. Firstly, we did not include a control sample from the general population or from non-dialysis patients with CKD. Secondly, the patients were not all vaccinated with the same number of doses and at the same time points relative to the serology assays, although this heterogeneity reflected care pathways and vaccination choices in a “real-life” population during an COVID-19 epidemic. However, the time interval between the third injection of vaccine and the most important serology assay (Ser_2_) was fixed (35 or 36 days). Thirdly, the study was performed in a single centre, and our findings may not necessarily extend to other geographical areas and other healthcare systems. Fourthly, the study participants did not undergo a serology assay immediately prior to the start of the study (e.g. in January 2021). However, all but one of the participants were seronegative for anti-S1 IgG in May 2020, and we considered the sole seropositive (but asymptomatic) patient to be a false positive. Fifthly, we did not perform a neutralization assay, which might have provided more information about the patients’ likely degree of protection from infection. However, the neutralization assay is technically complex and is not available routinely in medical biology laboratories.

In conclusion, our present data suggest that dialysis patients vaccinated with two doses of BNT162b2 might not have a sufficient level of protection against SARS-CoV-2 and should receive a third dose (at least) as part of a personalized vaccination strategy. We consider that due to (i) the emergence of new, virulent SARS-CoV-2 variants and (ii) the frequently reported post-fall in anti- SARS-CoV-2 antibodies some months after vaccination, the need for long-term, regular boosts with new or modified COVID-19 vaccines is likely for this patient population. Indeed, we have now started to recommend a fourth dose to our dialysis patients.

## Data Availability

The datasets used and/or analysed during the current study are available from the corresponding author on reasonable request.

## Abbreviations

AU: Arbitrary unit
BMI: Body mass index
CKD: Chronic kidney disease
COVID-19: Coronavirus disease 2019
IgG: Immunoglobulin G
IQR: Interquartile range
PCR: Polymerase chain reaction
S1: Spike
SARS-CoV-2: Severe acute respiratory syndrome coronavirus 2
SD: Standard deviation
